# Longitudinal associations between going outdoors and mental health and wellbeing during a COVID-19 lockdown in the UK

**DOI:** 10.1038/s41598-022-15004-0

**Published:** 2022-06-22

**Authors:** Sarah Stock, Feifei Bu, Daisy Fancourt, Hei Wan Mak

**Affiliations:** Department of Behavioral Science and Health, Institute of Epidemiology and Health Care, University College London, 1-19 Torrington Place, London, WC1E 7HB UK

**Keywords:** Psychology, Health care

## Abstract

The COVID-19 pandemic led to national lockdowns in countries around the world. Whilst lockdowns were shown to be effective in reducing the spread of disease, they were also associated with adverse effects on people’s mental health and wellbeing. Previous studies have suggested that time spent outside may have played a role in mitigating these negative effects, but research on this topic remains limited. Therefore, this study was designed to explore the longitudinal associations between going outdoors and people’s mental health and wellbeing during the first national lockdown (March–May 2020) in the UK. Data from 35,301 participants from the COVID-19 Social Study were analysed. Fixed effects regression was used to explore the longitudinal association between changes in going outdoors (the number of days spent outside) and changes in depressive symptoms, anxiety symptoms, life satisfaction and loneliness. A range of household and neighbourhood moderators were examined. Results show that an increase in the number of days spent outside was associated with decreases in depressive and anxiety symptoms and an increase in life satisfaction. Associations were more salient amongst people living with others, and those with greater satisfaction with their neighbourhood walkability and green spaces. No longitudinal association was found with loneliness. Overall, our analyses showed a positive association between going outdoors and improved mental health and wellbeing during the first COVID-19 lockdown in the UK. These findings are important for formulating guidance for people to stay well at home during pandemics and for the on-going nature-based social prescribing scheme.

## Introduction

The outbreak of coronavirus disease (COVID-19) was declared a pandemic by the World Health Organization on 11th March 2020. To slow the spread of the virus and reduce the burden on health services, many countries announced regional or national lockdowns. Whilst lockdowns are an effective tool in reducing the spread of disease, they can have a negative impact on people’s mental health and wellbeing, as shown through studies of lockdowns and quarantines in previous epidemics^1^, and echoed by a large number of studies conducted into the COVID-19 pandemic^2–5^. Recent research generally suggests there was a worsening in mental health during COVID-19 lockdowns both in the UK^3,6,7^ and on a global scale^8,9^.

In considering what it was about lockdowns during COVID-19 that led to this detrimental effect on mental health, a number of factors have been explored. Much of the research to date has focused on exploring pandemic-related stressors that occurred as a result of people being unable to leave their homes such as businesses having to make redundancies or furlough people leading to financial stressors for individuals, the closure of schools and placing new responsibilities on parents to home-school, an increase in domestic violence and abuse, and challenges accessing essentials such as food^10–12^. Many also suffered from illness due to COVID-19 or bereavement. All of these factors have been associated with poorer mental health and wellbeing during the pandemic^11^. In addition, the experience of being confined to the home was in itself a stressor. During the strict lockdown period in the UK, people could only leave their home once a day for essential reasons (e.g. exercise, food, medication). Such limitations on movement are fundamentally at odds to our innate human behaviours. Research has demonstrated the importance of sensory stimulation, novel experiences, and physical activity to our mental health and wellbeing^13–17^. Further, being outdoors is also important for human thriving; evidence from before the COVID-19 pandemic had already identified a positive impact of spending time outside on depression, anxiety and stress^18,19^. There are multiple reasons why being outdoors is thought to positively relate to improved mental wellbeing. For instance, the restorative characteristics provided by being outside, such as being away from a stressful environment, can aid recovery from mental exhaustion or fatigue^20,21^. One’s sense of vitality (physical and mental energy) can also be increased by spending time outside^22^, which is in turn associated with improved wellbeing^23^. If the outdoors engagement involves green space in particular, then this can also help reduce one’s exposure to harms such as air pollution, which can have adverse physical and mental health effects^24^.

To date, several studies have shown the importance that even limited time spent outdoors during COVID-19 lockdowns had on mental health. Evidence from a number of countries suggests better mental wellbeing and lower levels of some mental health difficulties amongst those people who spent more time outside during lockdowns^25–28^ and amongst those whose home or neighbourhood environment was greener^29^. A cross-sectional study of over 1000 people in the US found that people who spent more time outside during lockdown had lower odds of meeting depression criteria^30^. Research in Ireland using data from a single day in March 2020 found an association between time spent outdoors and reduced negative emotions and increased positive affect (how happy, relaxed and energetic participants were feeling)^31^. Other studies have focused on the benefits of outdoor activities during the pandemic such as gardening and exercising, which were shown to be positively related to mental health outcomes^32^; whilst other research has shown that resilience is greater amongst those going outside more often^33^. However, much of the existing research was based on cross-sectional data and tended to have small sample sizes. The relationship between mental health and wellbeing and time spent outside during COVID-19 lockdown is yet to be investigated using longitudinal analysis and a large sample.

Further, access to outdoor space and the associated mental health benefits were not equally available to everyone^34^. Some studies reported that people made the most of opportunities to be outside during lockdowns. For example, in Oslo, it was estimated that outdoor recreational activity, including visits to parks and green spaces, increased by 291% during the lockdown in March 2020 relative to the same days during the previous three years in 2017–2019^35^. Similarly, in the UK, a report of over 75,000 respondents found that people on average increased the number of days they went outside for 15 min or more from the start of the first lockdown in March 2020 to just before the lockdown was eased^36^. But, despite the increase, not everyone was able to enjoy time outside. Many people experienced limited access to good quality residential environments (such as places with good neighbourhood walkability) or expressed lower satisfaction with their neighbourhood, which limited their motivation to go outdoors and was associated with poorer self-rated health^37^, more depressive symptoms^38^, and lower life satisfaction^39^. This issue was particularly found amongst people from low income households and those from an ethnic minority background, who were also less likely to have access to private outdoor space (e.g. gardens or balconies, where time spent outside could be unlimited each day)^34,40,41^ and more likely to live in over-crowded households^42^. These groups were also affected disproportionately by the mental health implications of the COVID-19 pandemic^43^.

In this light, the present study was designed to explore the longitudinal association between changes in going outdoors and mental health and wellbeing throughout the first COVID-19 lockdown in the UK (23rd March to 11th May 2020). Additionally, given the uneven distribution of private and public outdoor spaces, we explored whether this longitudinal association was moderated by a range of household and neighbourhood factors. Understanding the associations between these factors is critical given that the impacts of the pandemic lockdowns on people’s mental health and wellbeing are likely to last for an extended period beyond the pandemic. Further, findings of this study may have relevance to (1) the new UK government pilot study into “Green Social Prescribing”, which has been implemented to understand the role of nature-based interventions on people’s mental health/wellbeing during and after COVID-19, and (2) nature-based social prescribing scheme more generally—one of the UK government’s and health sectors’ priorities in linking people to green space and nature-based interventions and activities to support mental wellbeing.

## Methods

### Participants

Data were drawn from the COVID-19 Social Study; a large panel study of the psychological and social experiences of over 70,000 adults (aged 18 +) in the UK during the COVID-19 pandemic. The study commenced on 21st March 2020 and involved online weekly and then monthly (24th August 2020 onwards) data collection from participants for the duration of the COVID-19 pandemic in the UK. The study did not use a random sample design and therefore the original sample is not representative of the UK population. But it does contain a heterogeneous sample that was recruited using three primary approaches. First, convenience sampling was used, including promoting the study through existing networks and mailing lists (including large databases of adults who had previously consented to be involved in health research across the UK), print and digital media coverage, and social media. Second, more targeted recruitment was undertaken focusing on (1) individuals from a low-income background, (2) individuals with no or few educational qualifications, and (3) individuals who were unemployed. Third, the study was promoted via partnerships with third sector organisations to vulnerable groups, including adults with pre-existing mental health conditions, older adults, carers, and people experiencing domestic violence or abuse. The study was approved by the UCL Research Ethics Committee [12467/005] and all participants gave informed consent. We confirm that the research was performed in accordance with relevant guidelines and regulations. The study protocol and user guide (which includes full details on recruitment, retention, data cleaning and sample demographics) are available at https://osf.io/jm8ra/.

In this study, we focused on participants who completed the study during the first UK national lockdown between 23rd March and 11th May 2020 (N = 65,727). Participants were restricted to those who had responded to the key mental health and wellbeing variables and control variables, as well as to those who had at least two survey responses during the observational period (N = 48,091). Participants who identified themselves as keyworkers or who reported leaving their homes to go to work were excluded from the analysis as they did not experience the same strictness of lockdown, this left an analytical sample size of 155,366 observations from 35,301 participants (4.4 per person, ranging from 2 to 8).

### Measures

#### Predictor

Going outdoors was measured using a single question on a scale of 0 to 7: “In the past 7 days, how many days have you been outside for 15 min or more (including on a balcony or in the garden)?”.

#### Outcome variables

Depressive symptoms were measured using the Patient Health Questionnaire (PHQ-9); a standard instrument for diagnosing depression in primary care^44^. The questionnaire involves nine items, with four-point responses ranging from “not at all” to “nearly every day”. Higher scores indicate greater levels of depression, ranging from 0 to 27.

Anxiety symptoms were measured using the Generalised Anxiety Disorder assessment (GAD-7); a well-validated tool used to screen and diagnose generalised anxiety disorder in clinical practice and research^45^. There are 7 items, with four-point responses range from “not at all” to “nearly every day”; higher scores indicate greater levels of anxiety, ranging from 0 to 21.

Life satisfaction was measured by a single question on a scale of 0 to 10: “overall, in the past week, how satisfied have you been with your life?”.

Loneliness was measured using the 3-item UCLA-3 Loneliness Scale, a short form of the Revised UCLA Loneliness Scale (UCLA-R)^46^. Each item is rated with a 3-point rating scale, ranging from “never” to “always”, with higher score indicating greater loneliness, ranging from 3 to 9.

#### Time-varying covariates

In our analysis, we considered five time-varying variables that might confound the association between going outdoors and mental health and wellbeing. These included *number of days respondents had face to face contact with others for* ≥ *15 min* in the past 7 days (including people living with respondents); *number of days respondents had phone/video call with others for* ≥ *15 min* in the past 7 days; *perceived social support* which was measured using an adapted version of the six-item short form of Perceived Social Support Questionnaire (F-SozU K-6)^47,48^ with higher scores indicating higher levels of perceived social support. Minor adaptations were made to the language in the scale to make it relevant to experiences during COVID-19 (see Supplementary Table S1 for a comparison of changes). Also included were *compliance with the government isolation guidance* measured using a single question on a 7-point scale, ranging from “not at all” to “very much so”, with higher score indicating higher compliance; and *self-isolation status* derived from answers to the question regarding current isolation status and reasons for isolating (including “I am self-isolating” and “I am worried about spreading COVID-19 to others”).

#### Potential household and neighbourhood moderators

We considered three household variables that may moderate the relationship between days spent outside and mental health/wellbeing. These included *living arrangement* (living alone vs living with others), *household overcrowding* defined as less than one room per person not including bathrooms or toilets (yes vs no), *access to garden and/or balcony* (yes vs no).

Additionally, we considered five neighbourhood variables that might be potential moderators. These included *living area* (rural area, small town, large town/city); *satisfaction with perceived walkability of neighbourhood* (satisfied vs neither/not satisfied); *access to green space* including a garden, balcony, small patio, roof terrace, a park, wood, or other green space within walking distance (yes vs no, none of these); *satisfaction with availability of usable green space/parks within neighbourhood* (satisfied vs neither/not satisfied); and *overall neighbourhood satisfaction* measured by a 5-point scale ranging from 0 “very dissatisfied” to 4 “very satisfied”.

### Analysis

Data were analysed using fixed-effects regression. This uses only within-individual variation, meaning that factors which do not vary over time (such as gender) are automatically accounted for. Compared to traditional regressions, this panel data method has the benefit of removing potential bias by controlling for both observed and unobserved individual heterogeneity. In this way it can be used to examine how the change in days spent outside was associated with mental health and wellbeing in individuals over time, independent of time-invariant factors^49^.

In the main analyses, fixed effects models were fitted separately for the four outcome variables, namely depressive symptoms, anxiety symptoms, life satisfaction and loneliness (UCLA-3), with days spent outside as the predictor controlling for time-variant covariates. Furthermore, moderation analyses were conducted individually to test whether the association between days spent outside and mental health/wellbeing differed by household and neighbourhood factors.

To account for the non-random nature of the sample, all analyses were weighted to the proportions of gender, age, ethnicity, education and country of living obtained from the Office for National Statistics^50^. The analyses were carried out using Stata/SE 16.1. The code to replicate the analyses is available at https://osf.io/6h2aj/.

### Ethics

The study was approved by the UCL Research Ethics Committee [12467/005] and all participants gave informed consent.

### PPI

The research questions in the UCL COVID-19 Social Study built on patient and public involvement as part of the UKRI MARCH Mental Health Research Network, which focuses on social, cultural and community engagement and mental health. This highlighted priority research questions and measures for this study. Patients and the public were additionally involved in the recruitment of participants to the study and are actively involved in plans for the dissemination of findings from the study.

## Results

### Descriptive

As shown in Table 1 within-individual variation accounted for around 39% of total variation in days spent outside, 16% in depressive and anxiety symptoms, 26% of variation in life satisfaction, and 18% in loneliness (Table 1). Demographic characteristics of the analytical sample are shown in the Supplementary Table S2.

**Table 1:** Descriptive statistics of days spent outside and mental health and mental wellbeing (N = 35,301; n = 155,366).

	**Days spent outside**	**Depressive symptoms**	**Anxiety symptoms**	**Life satisfaction**	**Loneliness**
Overall mean	4.63	6.64	5.06	5.77	4.95
Between-subject SD (σ_u_)	1.91	5.46	4.86	2.05	1.77
Within-subject SD (σ_e_)	1.54	2.35	2.15	1.22	0.83
Intraclass correlation (ρ)	0.61	0.84	0.84	0.74	0.82

### Depressive symptoms

After controlling for all time-invariant variables and important time-varying variables, the number of days spent outside was associated with a decreased number of depressive symptoms (coef = − 0.08, 95% CI = − 0.10, − 0.06) (Table 2; Supplementary Table S3 for the full table). When exploring the household and neighbourhood moderators, results showed that the association was more prominent for people who were satisfied with perceived walkability (coef = − 0.07, 95% CI = − 0.09, − 0.05 vs coef = − 0.01, 95% CI = − 0.06, 0.04), and for people who were satisfied with green space/parks (coef = − 0.07, 95% CI = − 0.09, − 0.05 vs coef = − 0.02, 95% CI = − 0.06, 0.03). No moderating effect was found for living arrangement, household overcrowding, garden/balcony access, living area, green space access, or overall neighbourhood satisfaction (Table 3).

**Table 2:** Fixed-effects models estimating the associations between days spent outside and mental health and mental wellbeing (N = 35,301; n = 155,366).

	**Depressive symptoms**			**Anxiety symptoms**			**Life satisfaction**			**Loneliness**		
	Coef	95% CI	*P* value	Coef	95% CI	*P* value	Coef	95% CI	*P* value	Coef	95% CI	*P* value
Number of days spent outside	**− 0.08**	**− 0.10, − 0.06**	**< 0.001**	**− 0.06**	**− 0.07, − 0.04**	**< 0.001**	**0.05**	**0.04, 0.06**	**< 0.001**	0.00	**− **0.00, 0.01	0.463

The models controlled for all time-invariant variables and time-varying variables including number of days respondents have had face to face contact with others for ≥ *15 min* in the past 7 days, number of days respondents have had phone/video call with others for ≥ *15 min* in the past 7 days, perceived social support, compliance with the government isolation guidance, and self-isolation status.

Significant values are in bold.

**Table 3:** Fixed-effects models estimating the associations between days spent outside and mental health and mental wellbeing: the moderating effects of household and neighbourhood factors.

	Depressive symptoms			Anxiety symptoms			Life satisfaction			Loneliness		
	Coef	95% CI	*P* value	Coef	95% CI	*P* value	Coef	95% CI	*P* value	Coef	95% CI	*P* value
**Household moderators**												
***Interacting with living arrangement (N* = *35,301; n* = *155,366)***												
Days spent outside	**− 0.08**	**− 0.10, − 0.06**	** < 0.001**	**− 0.07**	**− 0.09, − 0.06**	** < 0.001**	**0.05**	**0.04, 0.06**	** < 0.001**	0.00	**− **0.01, 0.01	0.855
Living alone * days spent outside	**− **0.00	**− **0.05, 0.04	0.843	**0.07**	**0.03, 0.10**	**0.001**	**− 0.03**	**− 0.05, − 0.00**	**0.016**	0.01	**− **0.01, 0.03	0.244
***Interacting with household overcrowding (N* = *35,301; n* = *155,366)***												
Days spent outside	**− 0.08**	**− 0.10, − 0.06**	** < 0.001**	**− 0.06**	**− 0.08, − 0.05**	** < 0.001**	**0.04**	**0.03, 0.05**	** < 0.001**	0.00	**− **0.00, 0.01	0.298
Household overcrowding*days spent outside	**− **0.01	**− **0.08, 0.06	0.819	0.03	**− **0.03, 0.09	0.287	0.03	**− **0.00, 0.06	0.062	**− **0.01	**− **0.03, 0.01	0.506
***Interacting with garden/balcony access (N* = *20,019; n* = *97,235)***												
Days spent outside	**− 0.11**	**− 0.17, − 0.05**	** < 0.001**	**− **0.00	**− **0.07, 0.06	0.984	**0.04**	**0.01, 0.07**	**0.005**	**− **0.00	**− **0.02, 0.02	0.791
Garden/balcony access*days spent outside	0.03	**− **0.03, 0.10	0.319	**− **0.07	0.13, 0.00	0.056	0.01	**− **0.02, 0.04	0.405	0.01	**− **0.02, 0.03	0.621
**Neighbourhood moderators**												
***Interacting with living area (N* = *35,301; n* = *155,366)***												
Days spent outside	**− 0.08**	**− 0.11, − 0.05**	** < 0.001**	**− 0.06**	**− 0.08, − 0.04**	** < 0.001**	**0.05**	**0.04, 0.06**	** < 0.001**	**− **0.00	**− **0.01, 0.00	0.423
Living area*days spent outside												
Small town	**− **0.00	**− **0.05, 0.04	0.918	0.01	**− **0.02, 0.05	0.447	0.00	**− **0.02, 0.02	0.942	0.01	**− **0.00, 0.03	0.131
Rural area	0.01	**− **0.04, 0.05	0.767	**− **0.01	**− **0.05, 0.02	0.491	**− **0.01	**− **0.03, 0.01	0.527	0.01	**− **0.00, 0.03	0.148
(REF: City/large town)												
***Interacting with perceived walkability satisfaction (N* = *17,659; n* = *87,235)***												
Days spent outside	**− **0.01	**− **0.06, 0.04	0.685	**− **0.00	**− **0.05, 0.05	0.975	**0.04**	**0.02, 0.07**	**0.002**	**− **0.02	**− **0.04, 0.01	0.186
Perceived walkability*days spent outside	**− 0.06**	**− 0.11, − 0.01**	**0.025**	**− 0.06**	**− 0.11, − 0.01**	**0.026**	**− **0.00	**− **0.03, 0.03	0.982	0.03	**− **0.00, 0.05	0.109
***Interacting with green space access (N* = *20,019; n* = *97,235)***												
Days spent outside	**− 0.07**	**− 0.09, − 0.04**	** < 0.001**	**− 0.05**	**− 0.08, − 0.03**	** < 0.001**	**0.05**	**0.03, 0.06**	** < 0.001**	0.01	**− **0.00, 0.02	0.277
Green space access*days spent outside	**− **0.03	**− **0.07, 0.01	0.142	**− **0.01	**− **0.05, 0.02	0.495	0.01	**− **0.02, 0.03	0.612	**− **0.01	**− **0.02, 0.01	0.219
***Interacting with green space/parks satisfaction (N* = *17,833, n* = *88,074)***												
Days spent outside	**− **0.02	**− **0.06, 0.03	0.545	**− **0.02	**− **0.06, 0.03	0.449	**0.04**	**0.01, 0.06**	**0.003**	**− **0.00	**− **0.02, 0.02	0.829
Green space/parks satisfaction*days spent outside	**− 0.06**	**− 0.11, − 0.00**	**0.039**	**− **0.04	**− **0.09, 0.01	0.094	0.01	**− **0.02, 0.03	0.663	0.00	**− **0.02, 0.03	0.694
***Interacting with overall neighbourhood satisfaction (N* = *17,845; n* = *88,123)***												
Days spent outside	**− **0.01	**− **0.08, 0.06	0.792	**− **0.00	**− **0.06, 0.06	0.997	**0.05**	**0.02, 0.09**	**0.005**	**− **0.05	**− **0.10, 0.00	0.070
Neighbourhood satisfaction * days spent outside	**− **0.02	**− **0.04, 0.00	0.114	**− **0.02	**− **0.03, 0.00	0.077	**− **0.00	**− **0.01, 0.01	0.576	**0.01**	**0.00, 0.02**	**0.047**

The models controlled for all time-invariant variables and time-varying variables including number of days respondents have had face to face contact with others for ≥ *15 min* in the past 7 days, number of days respondents have had phone/video call with others for ≥ *15 min* in the past 7 days, perceived social support, compliance with the government isolation guidance, and self-isolation status.

Significant values are in bold.

### Anxiety symptoms

For anxiety, the change in days spent outside was negatively associated with the change in anxiety symptoms (coef = − 0.06, 95% CI = − 0.07, − 0.04) (Table 2; Supplementary Table S3 for the full table). The association was stronger for people who lived with others (coef = − 0.07, 95% CI = − 0.09, − 0.06 vs coef = − 0.01, 95% CI = − 0.04, 0.03), as well as those who were satisfied with walkability in their neighbourhood (coef = − 0.06, 95% CI = − 0.08, − 0.04 vs coef = − 0.00, 95% CI = − 0.05, 0.05). No moderating effect was found for household overcrowding, garden/balcony access, living area, green space access, satisfaction with green space/parks within neighbourhood, or overall neighbourhood satisfaction (Table 3).

### Life satisfaction

Similarly, increases in days spent outside were associated with increased levels of life satisfaction (coef = 0.05, 95% CI = 0.04, 0.06) (Table 2; Supplementary Table S3 for the full table). Such association was more salient for people living with others (coef = 0.05, 95% CI = 0.04, 0.06 vs coef = 0.03, 95% CI = 0.01, 0.05). No moderating effect was found for other moderators (Table 3).

### Loneliness

Finally, no longitudinal association was shown between days spent outside and levels of loneliness (Table 2; Supplementary Table S3 for the full table), although there was some indication that the relationship between days spent outside and loneliness was moderated by neighbourhood satisfaction (coef = 0.01, 95% CI = 0.00, 0.02) (Table 3).

## Discussion

The current study was one of the first to use longitudinal analysis to examine the relationships between going outdoors and mental health and wellbeing during a COVID-19 lockdown, and to identify moderators that might influence the relationships. Fixed effects analysis revealed that an increase in the number of days spent outside was associated with decreases in depressive and anxiety symptoms and an increase in life satisfaction. No longitudinal association was found for days spent outside and loneliness. Further analysis revealed some moderating effects of household and neighbourhood factors. Specifically, if people felt satisfied with the “walkability” of their neighbourhood, their time spent outdoors was associated more strongly with lower anxiety and depression. If they felt satisfied with the local green space and parks, their time spent outdoors was associated more strongly with lower depression. Further, if they lived with other people, their time spent outdoors was more strongly associated with lower anxiety and greater life satisfaction. There was no evidence that the association between days spent outside and mental health/wellbeing differed by household overcrowding, garden/balcony access, living area, green space access and overall neighbourhood satisfaction.

Whilst our study is the first to consider the associations between spending time outside and improved mental health using large, longitudinal data in the context COVID-19 lockdowns, our findings are in line with previous research which has demonstrated associations both from before^51^ and during^30^ the COVID-19 pandemic. Such findings are supportive of the theory that spending time outside, such as in an urban park and in nature, promotes health, improves life satisfaction^52^ and can have restorative outcomes^53^. Further, the activities that people engage with outdoors such as physical activity and social interaction may also support wellbeing^54,55^. Specifically during the pandemic, increased time spent outside may also have counteracted screen-based activities such as watching TV, engaging with social media, and following the news on COVID-19 which were all found to be associated with an increased risk of depression and anxiety during lockdown^32^. In contrast, there was no evidence on the longitudinal association between days spent outside and loneliness (which has received less attention in previous studies). Notably, during the first lockdown, people were not permitted to meet with another person from outside their own household for their exercise, so being outdoors did not increase one’s specific social interactions. The findings presented here suggest that simply seeing other people outside (without interacting with them) was not sufficient to reduce loneliness, which echoes findings from previous psychological research^56^. However, it is possible that days spent outside was associated with social isolation. This remains to be explored further^57^.

Whilst our findings suggest improvements in mental health and wellbeing with an increase in going outdoors, our moderation analyses show that such relationships may differ depending on certain household and neighbourhood factors. For instance, we found that the associations between the number of days spent outside and reduced anxiety symptoms and improved life satisfaction were more prominent for people living with others than those living alone. However, it is notable that our analysis found no moderating effect of an overcrowded household on the association between days spent outside and mental health/wellbeing. This suggests that it was more than the increased feeling of space from being outside that meant time spent outdoors was associated with better mental health. Instead, it is possible that spending time outside may have provided an opportunity for people living with others to enjoy time being alone as a self-care strategy. Whilst some people experienced isolation during the pandemic, it was widely reported that others experienced the opposite: a lack of solitude^58^. Further, negative changes in relationships within families, increased breakdowns of relationships^59^, and more frequent incidents of domestic abuse during lockdown were cited during lockdowns^12,60^, which could have further exacerbated people’s mental health and wellbeing and increased people’s desire to have time alone. This is further supported by our findings that having access to a garden at home did not moderate the association between the changes in days spent outside and the changes in mental health/wellbeing. Instead, going outside of one’s home entirely into a different space appeared important to the relationship with mental health. Such findings are contrary to research from Spain during COVID-19 lockdowns, which found that the types of outdoor space accessible to individuals, such as private gardens, impacted the benefit of nature exposure on mental health outcomes^28^. Our findings may reflect the enriching nature of experiencing a novel or different environment by going outdoors, which is known to have rewarding properties for the brain^61,62^. In this way perhaps it is the change in scenery achieved by leaving one’s home and garden which is the most beneficial to mental wellbeing.

In relation to neighbourhood moderators, we found that the negative relationships between days outside and depression and anxiety appear to be stronger amongst people living in areas with greater satisfaction with perceived walkability and (for depression) greater satisfaction with green space/parks within the neighbourhood additionally moderated the relationship. These findings are in line with previous studies which show that people’s behaviours and health are influenced by such neighbourhood factors^63^; in particular, it has been shown that greater satisfaction with neighbourhood green space or walkability is associated with increased walking behaviour and better physical and mental health^55,64^. However, our results show no moderating effects of the availability of green space nor living area, inconsistent with findings from before the COVID-19 pandemic^19,54^. This is perhaps due to the benefits of a change of scenery regardless of one’s living area under extreme circumstances of a lockdown. Nonetheless, the findings above suggest that access to *good quality* green space/parks or a pleasant neighbourhood environment, rather than merely accessing any green space, is important in explaining some of the differences in mental health and wellbeing outcomes. Such findings have implications for urban planning and the importance of efficient built environment features which can heighten satisfaction with the walkability and green spaces within one’s neighbourhood. Features such as water landscaping, maximisation of green vegetation and trees, alongside the provision of wide, accessible footpaths and pavements can all work to make neighbourhood green space more satisfying and enjoyable to walk in^65,66^.

This study has a number of strengths, including its large sample size and use of weekly follow up over the entire seven weeks of the first national lockdown in the UK. While the UCL COVID-19 Social Study did not use random sampling, the study does have wide heterogeneity, including good stratification across all major socio-demographic groups. In addition, analyses were weighted on the basis of population estimates of core demographics, with the weighted data showing good alignment with national population statistics and another UK large scale nationally representative social survey^67^. However, we cannot rule out the possibility that the study inadvertently attracted individuals experiencing more extreme psychological experiences, with subsequent weighting for demographic factors failing to fully compensate for these differences. This study examined parallel longitudinal associations between days outside and mental health/wellbeing and therefore causality cannot be assumed. Future research is called to extend these findings to investigate the directionality of these relationships. Whilst the models controlled for all time-invariant variables and important time-varying variables and restricted to respondents who were not leaving the house to go to work, some relevant time-varying factors might have been omitted from the analysis (e.g. weather). Further, the main predictor for going outdoor was measured using a single item. They could be vulnerable to error variance relating to unclear substantive effects of the measures. Future study is encouraged to replicate this research paper by using alternative measures or combined measures that capture outdoor engagement in more detail. In relation to this, due to data unavailability, we were unable to examine whether people were going outside alone or with their household members. While our study considered people who had been outside for 15 min or more, the length of time people spent outdoors and the types of activities people engaged in when they were outside may also affect their wellbeing and these were not able to be considered with the measure in the current study. Future studies could consider the motivations, the length of time spent outdoors and whether people went out alone or with others when examining the longitudinal associations between going outdoors and mental health/wellbeing.

## Conclusions

Overall, our analyses revealed an association between an increased number of days outside and improved mental health and wellbeing during the first COVID-19 national lockdown in the UK. We found that such associations were strengthened for people living with others and people who were more satisfied with the perceived walkability of their area and its green spaces/parks. This study provides evidence for the importance of encouraging people to leave their homes even for limited exercise during lockdowns, and highlights the value of investing in high quality neighbourhood environments as a public health measure in town planning. The findings are particularly useful for the new government pilot study “Green Social Prescribing”, which has been designed to provide nature-based interventions to help improve mental health and wellbeing and to reduce health inequalities during and after COVID-19, as it suggests that such schemes have a theoretical basis for improving mental health that can now be tested further in intervention studies. The findings also support the implementation of broader nature-based social prescribing schemes beyond the pandemic to support mental wellbeing through connecting people to nature-based activities.

## Supplementary Information

Supplementary Tables.

## Data Availability

The data that support the findings of this study are available from UCL Covid-19 Social Study but restrictions apply to the availability of these data, which were used under license for the current study, and so are not publicly available. Data are however available from Dr Daisy Fancourt upon reasonable request and with permission of UCL Covid19 Social Study.
