# Supplementary material for: Longitudinal associations between going outdoors and mental health and wellbeing during a COVID-19 lockdown in the UK
Source: Sci Rep. 2022 Jun 22;12:10580. doi: 10.1038/s41598-022-15004-0 (PMC9216298; doi:10.1038/s41598-022-15004-0)
Supplement: Supplementary file 1 — Supplementary Tables. [file 41598_2022_15004_MOESM1_ESM.docx]

# Supplementary materials

| Supplementary Table S1: Comparison of items in the original and revised Perceived Social Support Questionnaire (F-SozU K-6). | |
| --- | --- |
| Original | Adapted for COVID-19  In the past week, I feel… |
| I experience a lot of understanding and security from others | I have experienced a lot of understanding and support from others |
| I know a very close person whose help I can always count on | I have a very close person whose help I can always count on |
| If necessary, I can easily borrow something I might need from neighbours or friends | If necessary, I can easily borrow something I need from neighbours or friends |
| I know several people with whom I like to do things | I have people with whom I can spend time and do things together |
| When I am sick, I can without hesitation ask friends and family to take care of  important matters for me | If I get sick, I have friends and family who will take care of me |
| If I am down, I know to whom I can go without hesitation | If I am feeling down, I have people I can talk to without hesitation |

| **Supplementary Table S2: Descriptive statistics of demographic characteristics and moderators (N= 35,301)** | | | |
| --- | --- | --- | --- |
|  | Variables | Percentages / mean (SD) | |
|  |  | Weighted | Unweighted |
| Age | 18-29 | 19.5% | 8.2% |
|  | 30-45 | 23.1% | 26.5% |
|  | 46-59 | 21.2% | 28.0% |
|  | 60+ | 36.2% | 37.3% |
| Gender | Women | 49.6% | 73.5% |
|  | Men | 50.4% | 26.5% |
| Ethnicity | White ethic | 87.3% | 95.6% |
|  | Ethnic minorities | 12.7% | 4.4% |
| Household income (N=31,802) | <30k | 50.7% | 41.3% |
|  | ≥30k | 49.3% | 58.7% |
| Education | Up to GCSE | 33.0% | 14.3% |
|  | A-level or equivalent | 33.4% | 17.5% |
|  | Degree or above | 33.7% | 68.2% |
| Living arrangement | Living alone | 19.1% | 20.1% |
|  | Living with others | 80.9% | 79.9% |
| Household overcrowding | Yes | 16.2% | 10.4% |
|  | No | 83.8% | 89.6% |
| Access to garden/balcony (N=20,019) | Yes | 77.5% | 77.2% |
|  | No | 22.5% | 22.8% |
| Living area | City/large town | 54.0% | 50.5% |
|  | Small town | 24.3% | 24.8% |
|  | Rural location | 21.7% | 24.7% |
| Satisfaction with perceived walkability of neighbourhood (N=17,659) | Satisfied | 83.6% | 85.9% |
|  | Not satisfied | 16.4% | 14.1% |
| Access to green space (N=20,019) | Yes | 48.6% | 50.0% |
|  | No | 51.4% | 50.0% |
| Satisfaction with availability of usable green space/parks within neighbourhood (N=17,833) | Yes | 80.1% | 83.2% |
|  | No | 19.9% | 16.8% |
| Overall neighbourhood satisfaction (5-point scale, ranging from 0 “very dissatisfied” to 4 “very satisfied”) (N=17,845) |  | 2.03 (.905) | 2.12 (.877) |

| **Supplementary Table S3: Fixed-effects models estimating the associations between days spent outside and mental health and mental wellbeing, including coefficients for time-varying covariates (N=35,301; n=155,366)** | | | | | | | | | | | | | |
| --- | --- | --- | --- | --- | --- | --- | --- | --- | --- | --- | --- | --- | --- |
|  | **Depressive symptoms** | | | **Anxiety Symptoms** | | | **Life Satisfaction** | | | | **Loneliness** | | |
|  | Coef | 95% CI | P-value | Coef | 95% CI | P-value | Coef | 95% CI | P-value | Coef | | 95% CI | P-value |
| Number of days spent outside | -0.08 | -0.10, -0.06 | <0.001 | -0.06 | -0.07, -0.04 | <0.001 | 0.05 | 0.04, 0.06 | 0.000 | 0.00 | | 0.00, 0.01 | 0.463 |
| Number of days with face to face contact with others | 0.02 | 0.01, 0.04 | 0.006 | 0.00 | -0.01, 0.02 | 0.743 | 0.01 | 0.00, 0.01 | 0.159 | 0.00 | | -0.01, 0.01 | 0.872 |
| Number of days video called with others | -0.01 | -0.03, 0.01 | 0.159 | 0.01 | -0.01, 0.03 | 0.233 | 0.02 | 0.01, 0.03 | <0.001 | -0.01 | | -0.01, 0.00 | 0.025 |
| Perceived social support | -0.10 | -0.11, -0.09 | <0.001 | -0.07 | -0.08, -0.06 | <0.001 | 0.05 | 0.05, 0.06 | <0.001 | -0.04 | | -0.05, -0.04 | <0.001 |
| Compliance with government isolation guidance | 0.04 | -0.03, 0.12 | 0.264 | 0.00 | -0.05, 0.06 | 0.938 | 0.04 | 0.01, 0.08 | 0.021 | -0.02 | | -0.04, 0.00 | 0.067 |
| Self-isolation status | -0.07 | -0.15, 0.01 | 0.078 | -0.40 | -0.48, -0.32 | <0.001 | 0.05 | 0.01, 0.08 | 0.016 | 0.00 | | -0.02, 0.03 | 0.794 |
